# BRI DataLab: an AI-assisted research platform for Chinese infrastructure finance

**DOI:** 10.3389/fdata.2026.1863692

**Published:** 2026-08-27

**Authors:** Sanoop Sajan Koshy

**Affiliations:** Department of Humanities and Social Sciences, Indian Institute of Technology Madras„ Chennai, Tamil Nadu, India

**Keywords:** Belt and Road Initiative, Chinese development finance, computational social science, digital methods, research platform, retrieval-augmented generation

## Abstract

BRI DataLab is an open-source web platform that integrates a harmonized infrastructure financing dataset, a curated policy and academic document corpus, and a retrieval-augmented generation (RAG) interface into a single queryable research environment for Chinese overseas development finance. Analysts working on the Belt and Road Initiative (BRI) must move between datasets, government documents, and academic literature without an integrated interface for querying across all three at once. This paper documents BRI DataLab, a research platform that combines a harmonized project-level dataset derived from AidData's China's Global Loans and Grants Dataset v1.0 with a curated corpus of 42 policy documents, institutional reports, and academic sources, accessed through an AI-assisted retrieval and synthesis layer built on RAG. Three methodological contributions are described: the decisions involved in converting more than 33,000 tranche-level financing records into 4,861 infrastructure projects; the four-category selection framework governing the document collection; and the system design of the AI interface, including the epistemic constraints built into its system prompt. The central argument is that AI-assisted research interfaces are methodologically defensible when they are transparent about what they can and cannot establish.

## 1 Introduction

The study of Chinese overseas infrastructure finance has an infrastructure problem of its own. The most comprehensive public record of Chinese official overseas lending, AidData's China's Global Loans and Grants Dataset (AidData, 2025), contains over 33,000 tranche-level financing records spanning more than 160 countries and two decades. The key policy documents governing BRI financing, from the Ministry of Finance's Guiding Principles to the Belt and Road Forum communiqués (Ministry of Finance, People's Republic of China, 2017, 2019), are publicly available but scattered across government portals. The peer-reviewed scholarship on these patterns, covering debt sustainability, governance conditionality, the contested debt-trap thesis, and regional political economy, spans multiple disciplines and runs to thousands of papers. No integrated interface has brought these three layers together in a way that lets researchers move between what China committed, what official documents say about those commitments, and what scholarship has established about their implications.

Existing tools for Chinese development finance research operate on single data layers. AidData's online portal provides project-level search and filtering but no document retrieval or AI synthesis. Standard academic search engines retrieve literature but cannot query structured financing data. General-purpose AI assistants (LLMs) draw on training data without grounding responses in curated, domain-specific sources, producing outputs that are unverifiable and difficult to cite. To our knowledge, no published platform integrates structured financing data, a curated policy and academic document corpus, and a constrained AI synthesis layer into a single queryable interface for BRI or Chinese development finance research. BRI DataLab fills this gap.

BRI DataLab integrates three components: a harmonized project-level infrastructure financing dataset derived from AidData's China's Global Loans and Grants Dataset v1.0, a curated corpus of 42 documents spanning official Chinese government policy papers, multilateral institutional reports, and peer-reviewed academic literature, and an AI-assisted retrieval and synthesis layer built on retrieval-augmented generation (RAG). The platform covers 183 countries globally and has particular depth on South Asia, where Pakistan, Sri Lanka, Bangladesh, Myanmar, Nepal, and the Maldives together account for more than $110 billion in Chinese infrastructure financing commitments. The platform is publicly accessible at https://bri-datalab.streamlit.app, with source code available at https://github.com/sanoop-ssk/bri-datalab.

This paper documents the methodological decisions behind the tool, not its interface design. Three contributions are described. First, converting tranche-level AidData records into a clean project-level infrastructure dataset required a series of harmonization choices about aggregation logic, country classification, and title standardization that have direct consequences for how the data can and cannot be used. Second, the document corpus was built on explicit inclusion criteria across four source categories, and those criteria determine what the AI system can responsibly retrieve and synthesize. Third, the AI system design, particularly the epistemic constraints built into its system prompt, reflects a deliberate position on what AI-assisted synthesis can legitimately do in IR research. As Linegar et al. (2023) note, large language models bring genuine analytical opportunities to political science research alongside significant methodological risks. The design choices documented here represent one approach to managing that tension. This paper contributes a domain-specific AI-assisted research platform that integrates structured development finance data with curated documentary sources through a constrained retrieval-augmented generation architecture.

## 2 System components and data design

### 2.1 Platform overview

BRI DataLab is a web-based research platform with three integrated layers accessible through a single Streamlit interface. The Chat interface accepts natural language queries and routes them through a four-layer decision system before retrieving responses from the financing dataset, the document corpus, or both. The Data Explorer provides direct structured access to the financing data through interactive filters, charts, and a project-level table without any AI mediation. The Document Library displays the four-category corpus with coverage metadata and inclusion criteria.

**Figure 1:**
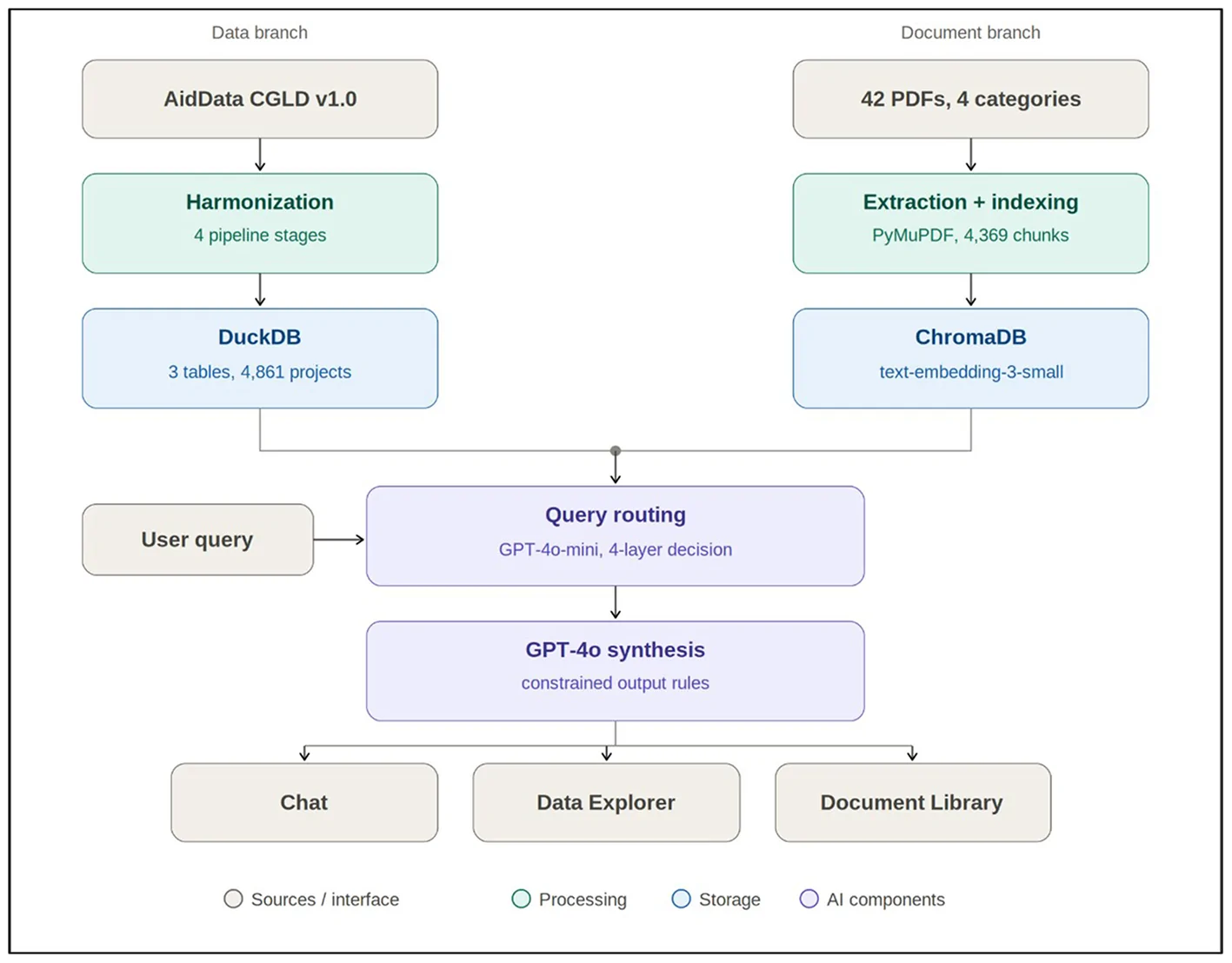
End-to-end architecture of BRI DataLab. The system connects two data ingestion pipelines (AidData harmonization → DuckDB; PDF corpus → ChromaDB) to a multi-layer query routing system, which dispatches queries to structured data retrieval, semantic document retrieval, or both. GPT-4o handles synthesis; GPT-4o-mini handles classification and routing. All user interaction occurs through a Streamlit web interface with three modules: Chat, Data Explorer, and Document Library.

The platform connects two backend data stores: a DuckDB relational database containing 4,861 harmonized infrastructure projects and a ChromaDB vector store containing 4,369 embedded document chunks. Query routing and response synthesis are handled by GPT-4o and GPT-4o-mini via the OpenAI API, orchestrated through LlamaIndex. Figure 1 shows the end-to-end system architecture.

### 2.2 Financing data pipeline

The harmonization pipeline runs in four sequential stages: (1) sector filtering to retain infrastructure-relevant records; (2) tranche-to-project aggregation via Parent_ID grouping; (3) co-funder classification to separate Chinese official finance from commercially co-financed records; and (4) currency standardization to 2023-constant USD. An optional preprocessing step applies a GPT-4o-mini title-cleaning pass to standardize display names without altering substantive fields. Table 1 documents each stage with its input, output, and rationale.

**Table 1:** Data harmonization decisions.

Decision	Input	Output	Rationale
Infrastructure sector filter	33,580 tranche-level records	Infrastructure-sector records only	Excludes budget support, scholarships, and technical assistance; retains energy, transport, communications, water supply, and industry/mining/construction
Tranche-to-project aggregation	Multi-tranche records (via Parent_ID)	Project-level consolidated records	Prevents single projects from appearing as multiple records, distorting counts, and financial totals
Developed-country co-funder flag	All records	Separation into bri_projects_core (4,498 rows) and bri_projects_full (4,861 rows)	Distinguishes Chinese official finance from records where Western commercial banks participated as co-financiers
Currency standardization	Amount_Nominal_USD	Amount_Constant_USD_2023 as primary analytical metric	Enables cross-year financial comparison without inflation distortion; nominal figures retained for reference
Title cleaning pass	Inconsistent, truncated, or transliterated project display names	Standardized Display_Title field	Improves readability and searchability without altering any substantive data field

Source: author's data pipeline applied to AidData (2025).

The primary data source is AidData's China's Global Loans and Grants Dataset, Version 1.0 (AidData, 2025), based on the TUFF 4.0 methodology (Parks et al., 2025), the most comprehensive public record of Chinese official overseas finance available. In its full form, the dataset contains 33,580 tranche-level records covering official finance flows from Chinese government entities to 165 recipient countries between 2000 and 2023. Using this dataset directly for BRI infrastructure research raises two problems. The tranche-level structure means a single project financed across multiple disbursements appears as multiple records, inflating project counts, and distorting financial totals. And the full dataset includes financing types, budget support, technical assistance, scholarships, and debt relief, that are analytically inappropriate for studies focused on physical infrastructure and BRI-related capital formation.

Other publicly available initiatives also document Chinese overseas development finance using different methodological approaches. The Boston University Global Development Policy Center's *China's Overseas Development Finance Database* (Gallagher and Ray, 2020) focuses primarily on lending by China's policy banks, while Horn et al. (2021) reconstruct sovereign lending using debt-stock estimates and balance-sheet evidence. These resources provide complementary perspectives on Chinese overseas finance but differ from AidData in coverage, unit of analysis, and methodological design. BRI DataLab adopts AidData's TUFF 4.0 dataset because its tranche-level project records, standardized sector classifications, and detailed project metadata are particularly well suited to the harmonization pipeline presented in Table 1. This choice reflects methodological suitability for project-level infrastructure analysis rather than a claim that any single dataset provides a complete representation of Chinese overseas finance.

A data harmonization pipeline addresses both problems through a sequence of decisions summarized in Table 1.

The infrastructure filter retains records in sectors involving direct physical capital formation: energy, transport and storage, communications, water supply and sanitation, and industry, mining, and construction. The tranche-to-project aggregation groups filtered records by their AidData Parent_ID field, combining all financing tranches for a single project into one consolidated record. All financial calculations use Amount_Constant_USD_2023, which is 2023-constant dollars, so comparisons across years are not varied by inflation. Nominal figures are retained in the database for reference but are not used in analytical outputs.

Two additional decisions address specific data quality concerns. A developed-country flag identifies records where Western commercial banks participated alongside Chinese state entities as co-financiers. Including these records in an analysis of Chinese official finance blends analytically distinct financing modalities. The core analytical table, bri_projects_core with 4,498 rows, excludes such records; the full table bri_projects_full with 4,861 rows retains them. A title cleaning pass is done by GPT-4o-mini model to standardize project display names, which in the original AidData records were frequently inconsistent, truncated, or in transliterated Chinese, without altering any substantive field.

Approximately 19% of records have undisclosed commitment amounts, which means financial totals systematically undercount the true volume of Chinese infrastructure finance. For some recipient countries the proportion of projects with undisclosed amounts runs considerably higher, so country-level totals should be read as lower bounds rather than definitive figures. The dataset covers official finance only, while private foreign direct investment and informal financing flows fall entirely outside its scope. For instance, the CPEC case illustrates this gap. The $62 billion figure commonly cited for CPEC includes announced project packages and private investment that AidData's methodology does not capture. All figures represent financing commitments, not disbursements. A commitment records an agreement to finance a project. Whether funds actually moved, at what pace, and in what form requires separate verification against project-level implementation records that AidData does not provide. BRI DataLab makes this distinction explicit in every financial response.

### 2.3 Document corpus architecture

The document corpus consists of 42 documents saved as PDFs indexed for AI retrieval. The corpus indexing pipeline runs in three stages. First, text is extracted from each PDF using PyMuPDF, with URL artifacts and metadata headers removed during a filtering pass. Second, the cleaned text is chunked into segments of approximately 1,400 characters with overlap, producing 4,369 chunks across the 42 documents. Third, each chunk is embedded using OpenAI's text-embedding-3-small model and stored in a ChromaDB persistent collection named bri_documents. The collection is pre-built and hosted on Hugging Face (sanoop-ssk/bri-monitor-chromadb), downloaded automatically on first deployment. Source filenames are mapped to proper institutional citation names through a lookup dictionary at retrieval time, so the model never returns filename-style references in its output.

Documents were selected through a four-category framework designed to cover the source types most relevant to BRI finance research, with different inclusion criteria for each category reflecting its source type (Table 2).

**Table 2:** Document corpus composition.

Category	Description	Documents	Coverage period	Inclusion criteria
1. Official Chinese Government Policy	BRI white papers, MoF financing guidelines, NDRC/MFA/MOFCOM frameworks, green and energy cooperation frameworks	10	2015–2023	Comprehensive: all available English-language BRI financing policy documents from Chinese ministries and agencies
2. Official Speeches & Forum Communiqués	Xi Jinping BRF keynote speeches; BRF joint communiqués; BRF working group communiqués	6	2017, 2019, 2023	Comprehensive: all three Belt and Road Forum events represented
3. Multilateral & Institutional Reports	World Bank, IMF, AidData, UN, Green Finance & Development Center (GFDC) reports	12	2019–2026	Comprehensive within scope: reports directly addressing BRI financing, debt sustainability, or Chinese development finance from institutions with established methodological frameworks
4. Academic & Research Literature	Peer-reviewed articles and research institution reports	14	2018–2024	Selective: Q1/Q2 journals or established institutions; directly addressing Chinese overseas infrastructure finance or BRI debt; 50+ citations or recognized BRI scholars
Total		**42**	**2015–2026**	

Bold values indicate the total number of documents in the corpus and the overall coverage period.

For Category 1, no quality filter was applied to Chinese government documents because these are primary sources documenting China's official BRI framing, including the National Development and Reform Commission (NDRC), Ministry of Foreign Affairs (MFA), and Ministry of Commerce (MOFCOM) Vision and Actions (NDRC, MFA, & MOFCOM, 2015), the Ministry of Finance Guiding Principles on Financing the Development of the Belt and Road (2017), the BRI Debt Sustainability Framework (2019), the Green Belt and Road Guidance, and the Energy Cooperation Vision. These documents are not treated as independently validated empirical evidence, but as statements of official policy positions. This makes the distinction between what these sources assert and what scholarship independently supports analytically central, and that distinction is enforced by the system prompt (see Section 3.3).

For Category 2, speeches and forum communiqués are included as authoritative statements of China's official policy articulation at key Belt and Road Forum events. These documents provide insight into leadership-level framing and evolving strategic priorities, rather than operational or project-level evidence, and are interpreted accordingly within the system.

Category 3 covers institutional reports, including the Green Finance & Development Center's (GFDC) BRI Investment Reports (Nedopil, 2024, 2026), the World Bank's Belt and Road Economics (World Bank, 2019), AidData's Banking on the Belt and Road (Malik et al., 2021), How China Lends (Gelpern et al., 2021), How China Collateralizes (Gelpern et al., 2025), the BRI-SDGs Progress Report (United Nations, 2022), and IMF and World Bank debt sustainability assessments. The GFDC reports are particularly important as they extend coverage of BRI investment trends beyond AidData's 2023 dataset.

Category 4 was curated against explicit criteria: publication in Q1 or Q2 journals or by established research institutions; direct engagement with Chinese overseas infrastructure finance or BRI debt; and either 50 or more citations or authorship by recognized scholars in the field. The current corpus prioritizes scholarship most directly aligned with the platform's methodological objectives, particularly studies on Chinese overseas infrastructure finance, BRI debt, and AidData's measurement framework. This provides broad coverage of the literature required to support the platform's current analytical functions, while recognizing that it does not comprehensively represent every strand of scholarship on Chinese overseas development finance. Future versions of the platform will expand the corpus to incorporate additional perspectives on sovereign lending, development finance, political economy, and legal governance.

The document corpus has one structural limitation; it is English-language only. Chinese-language primary documentation, including project contracts, inter-ministerial directives, and provincial investment plans, contains information about BRI implementation that does not appear in the English-language corpus. BRI DataLab cannot address research questions that require this material, and this limitation is stated explicitly in the platform interface.

## 3 System architecture and implementation

### 3.1 System overview and data flow

BRI DataLab's AI layer runs on LlamaIndex's retrieval-augmented generation (RAG) framework, connecting two data stores: a DuckDB relational database for structured financing records and a ChromaDB vector store for the document corpus. GPT-4o is used for SQL generation, data interpretation, and hybrid synthesis, tasks that require depth and judgment. GPT-4o-mini handles topic classification, query routing, and follow-up question generation, where speed matters more than interpretive precision. The full system runs as a Streamlit web application. Figure 1 shows the end-to-end system architecture. The pipeline has two distinct ingestion branches—structured data and unstructured documents, that converge at the query routing layer before synthesis.

RAG addresses a core limitation of general-purpose language models in domain-specific research, that is, their tendency to produce confident, plausible-sounding claims in areas where their training data is limited, contested, or outdated (Lewis et al., 2020). For BRI research, where empirical claims are frequently contested across methodological and ideological lines, as the decade-long debate over debt-trap diplomacy shows (Brautigam, 2020; Jones and Hameiri, 2020), grounding responses in specific retrieved source passages is not merely a technical preference. It is an epistemic requirement.

### 3.2 Query classification and routing

**Figure 2:**
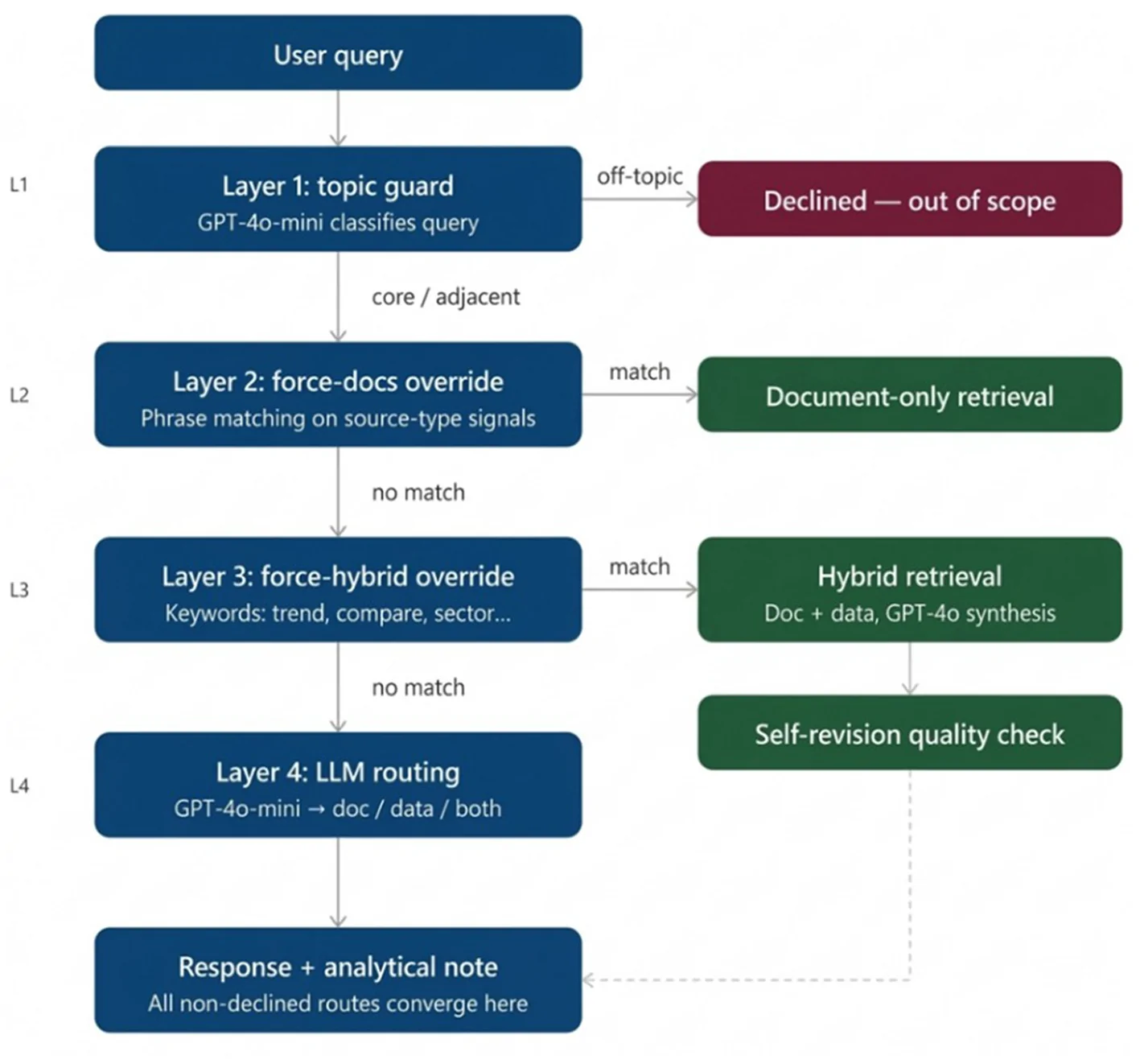
Query routing architecture in BRI DataLab. Each query passes through four sequential decision layers before retrieval and synthesis. Layers 2 and 3 are rule-based overrides; LLM-based routing at Layer 4 applies only when neither override is triggered.

Every query passes through a four-layer decision process before any retrieval occurs. Figure 2 shows this routing logic.

The first layer is an off-topic guard. LLM classifies and identifies each incoming query as core (BRI or Chinese infrastructure finance), adjacent (IR theory, development economics, or geopolitics broadly), or off-topic (unrelated to IR or development finance). Off-topic queries are declined with no retrieval attempted. Adjacent queries proceed but carry a flag that instructs the retrieval step to note corpus limitations explicitly.

The second layer is a force-documents override. Queries matching defined patterns, including references to white papers, BRI Forum speeches, Xi Jinping statements, guiding principles, or debt sustainability frameworks, route directly to document-only retrieval, bypassing LLM routing entirely. This prevents document-content questions from getting mixed up with data interpretation.

The third layer is a force-hybrid override. A set of keywords including trend, sector, compare, vs. policy, narrative, current, recent, and any year beyond the data limit triggers mandatory hybrid routing regardless of what LLM routing would otherwise decide. The logic is straightforward: these query types need both what the data shows and what scholarship contextualizes. Neither the dataset nor the corpus alone gives a complete answer.

The fourth layer is default LLM routing, which applies to queries not caught by the preceding three. LLM classifies these as documents, data, or both, with both as the fallback when the classification is ambiguous.

For hybrid queries, synthesis is followed by a self-revision quality check. LLM reviews the draft response against five criteria: relevance to the actual question asked; numerical accuracy against the data returned; absence of vague filler language; absence of causal claims without quantitative support; and country-level specificity when named countries appear in the query. The draft is rewritten if problems are found.

A dedicated mechanism handles post-2023 queries. When a query references years 2024 to 2026, or terms such as “current” or “ongoing,” the synthesis prompt directs GPT-4o to draw on the GFDC's BRI Investment Reports for post-2023 context (Nedopil, 2024, 2026), attribute each claim to its source, and state explicitly where dataset coverage ends. This provides a built-in recognition of the system's temporal boundary.

### 3.3 Output constraint design

The most consequential design decisions in BRI DataLab concern what the system prompt prohibits rather than what it enables. The system prompt contains eight epistemic rules, five of which carry direct analytical weight.

(1) **Source-only claims**. The AI may only include claims directly supported by a retrieved source passage or an AidData query result provided to it in that session. It may not draw on general training knowledge to fill gaps. When sources are insufficient, it must say so.
(2) **Attribution of contested claims**. Three categories of empirical claims must always be attributed to a specific named source and flagged as contested; not stated as descriptive fact, such as, claims about governance or corruption profiles that Chinese finance targets; claims about strategic motivations behind financing decisions; and claims comparing Chinese and Western financing models. The required framing is: “According to [source], scholars argue that...” These are among the most empirically contested questions in BRI scholarship, and the system must not present that contestation as settled.
(3) **Commitment/disbursement distinction**. Every response involving AidData figures must note that those figures represent financing commitments, not disbursements. This note is mandatory.
(4) **Causal language prohibition**. Causal claims are prohibited unless they come from a cited quantitative study. The system uses “associated with” and “coincides with” rather than “caused” or “led to.”
(5) **Mandatory analytical note**. Every response ends with an analytical note stating what the analysis establishes from the sources, what it cannot establish, and what further verification is recommended. This is not a standard disclaimer. It is the mechanism for preserving the distinction between exploration and finding, which Grimmer and Stewart (2013) identify as central to the responsible use of automated methods in political science.

### 3.4 Interface modules

Alongside the Chat interface, BRI DataLab includes a Data Explorer that gives direct structured access to the financing dataset without AI mediation. Researchers can filter by region, sector, flow type, and time period manually, and also using certain quick-filter presets. Results are displayed as a proportional symbol map, analytical charts covering financing trends, sectoral distribution, and regional comparison, or a project-level data table with all key fields visible. Summary statistics update dynamically with each filter across the coverage period.

The Document Library interface displays the four corpus categories with coverage periods and inclusion criteria, and provides a notice that the Research Assistant draws exclusively from this indexed corpus. This transparency layer allows users to assess the evidentiary basis of any AI-generated response before using it in their work.

### 3.5 System specifications

BRI DataLab connects several open-source and commercial components, each selected for a specific functional role. DuckDB was chosen for its in-process analytical query performance on structured tabular data without requiring a separate database server. ChromaDB provides a lightweight persistent vector store compatible with Streamlit's deployment environment. Temperature settings for LLM calls are set conservatively—zero for routing and SQL generation, where determinism matters, and 0.1 for synthesis, where a small amount of variation improves response fluency without introducing instability. Table 3 summarizes the full component stack and key operational parameters.

**Table 3:** BRI DataLab system specifications and key parameters.

Component	Specification
Application framework	Streamlit 1.35.x, Python 3.11
Structured data store	DuckDB 0.10.x (read-only, in-process)
Vector store	ChromaDB 0.5.x, persistent client
Embedding model	OpenAI text-embedding-3-small (1,536 dimensions)
Retrieval parameter	similarity_top_k = 15, chunk size = 1,400 characters
Synthesis model	GPT-4o (temperature 0.1 for synthesis, 0 for SQL)
Routing/classification model	GPT-4o-mini (temperature 0 for routing)
Document corpus	42 PDFs, 4,369 text chunks after preprocessing
Dataset	4,861 projects, 3 DuckDB tables, 183 countries
Deployment	Streamlit Community Cloud; ChromaDB downloaded from Hugging Face on first run
Approximate latency	8–20 s under typical query conditions*^***a***^*

Source: author's implementation. ^**a**^Complex multi-layer analytical queries may require longer depending on retrieval complexity and synthesis workload.

All components are open-source except the OpenAI models used for query routing and response synthesis, which currently require access through the OpenAI API. This dependency currently limits complete computational reproducibility because the AI-assisted reasoning layer relies on proprietary models.

## 4 System demonstration and validation

The three demonstrations below validate the four-layer routing architecture described in Section 3.2. Each use case targets a different routing path: Use Case 1 tests the force-hybrid override triggered by sector-type keywords; Use Case 2 tests the force-documents override triggered by source-type phrase patterns; Use Case 3 tests multi-keyword hybrid routing for comparative queries. For each, the query submitted is shared, the routing decision taken, the system output, what the response establishes, and what it cannot establish.

### 4.1 Sector-level query validation (Use Case 1)

*User query:* “What are the top sectors by Chinese infrastructure financing commitments in South Asia, and which countries received the most in each sector?”

The term “sectors” triggered the force-hybrid override, routing the query to both the bri_projects_south_asia table and the document collection. The AidData results identify Energy and Transport and Storage as the dominant sectors, with Pakistan receiving the largest commitments in both. Other countries, including Bangladesh, India, and Sri Lanka, also feature prominently but at lower levels. By contrast, Communications remains comparatively small across the region.

The hybrid retrieval layer complemented the quantitative results with contextual interpretation while preserving clear source boundaries. The analytical note correctly distinguished what could be established directly from the AidData records from what would require additional documentary evidence. Rather than generating speculative explanations for sectoral differences, the system explicitly identified the limits of the indexed corpus and recommended further investigation where appropriate. This behavior demonstrates one of the platform's central design objectives: supporting exploratory analysis without extending beyond the available evidence.

**Figure 3:**
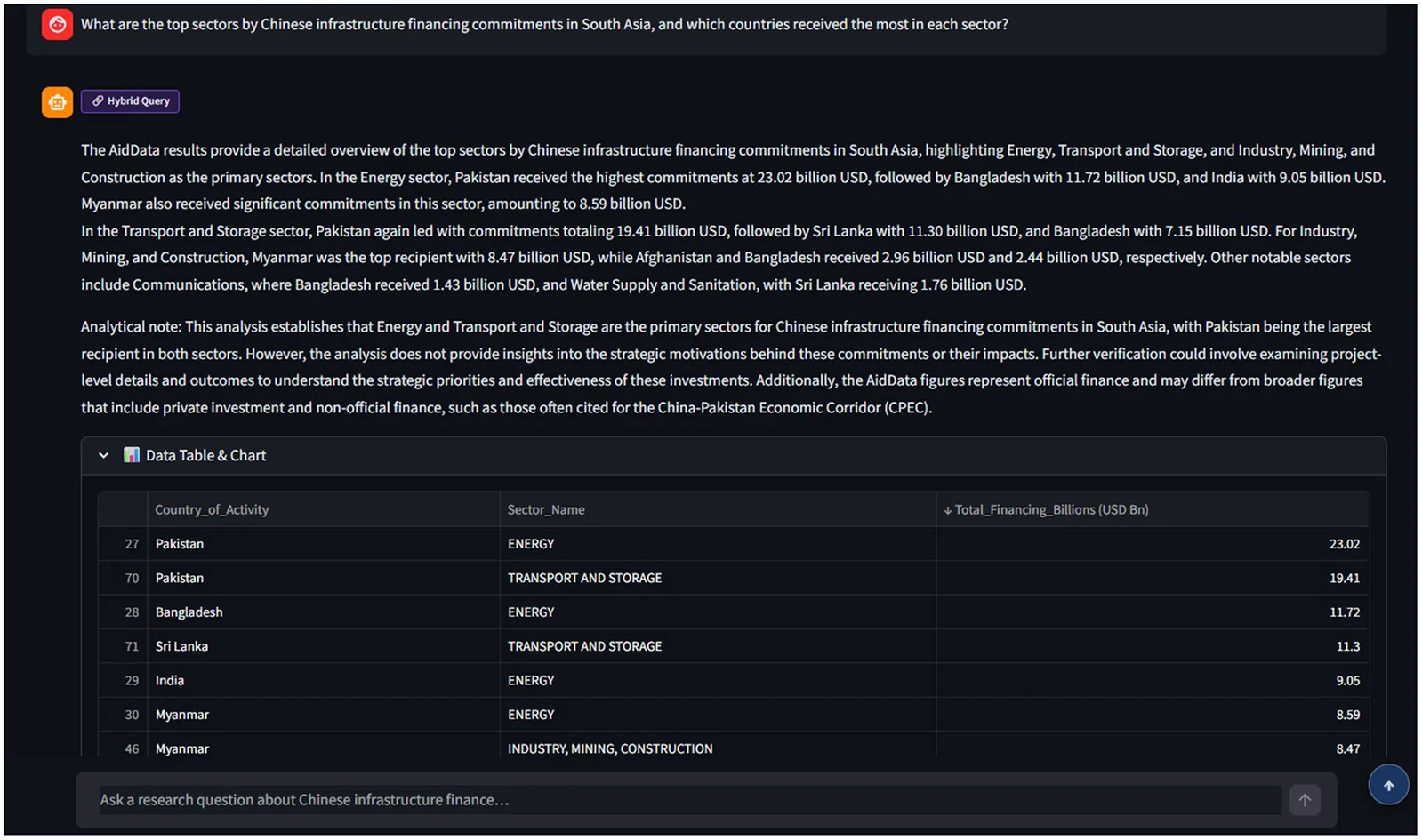
Use Case 1: query + response.

This output (Figure 3) confirms that the force-hybrid override routes sector-type queries correctly and that the analytical note mechanism functions as designed, identifying corpus gaps rather than generating unsupported narrative.

### 4.2 Document retrieval validation (Use Case 2)

*User query:* “What do Chinese government white papers and BRI Forum documents say about the guiding principles for BRI financing?”

The phrase “white papers” matched the force-documents override, routing this query directly to document-only retrieval from the indexed sources. The response drew on the Ministry of Finance Guiding Principles (2017), the Ministry of Finance's BRI Debt Sustainability Framework (2019), the Belt and Road Forum communiqués, and the World Bank's Belt and Road Economics (2019). It synthesized the official Chinese framing around transparency, sustainability, and mutual benefit, attributed the “Open and Clean BRI” initiative to its documentary sources rather than asserting it as independent fact, and credited the Ministry of Finance document specifically for the principles on financial innovation and risk management.

**Figure 4:**
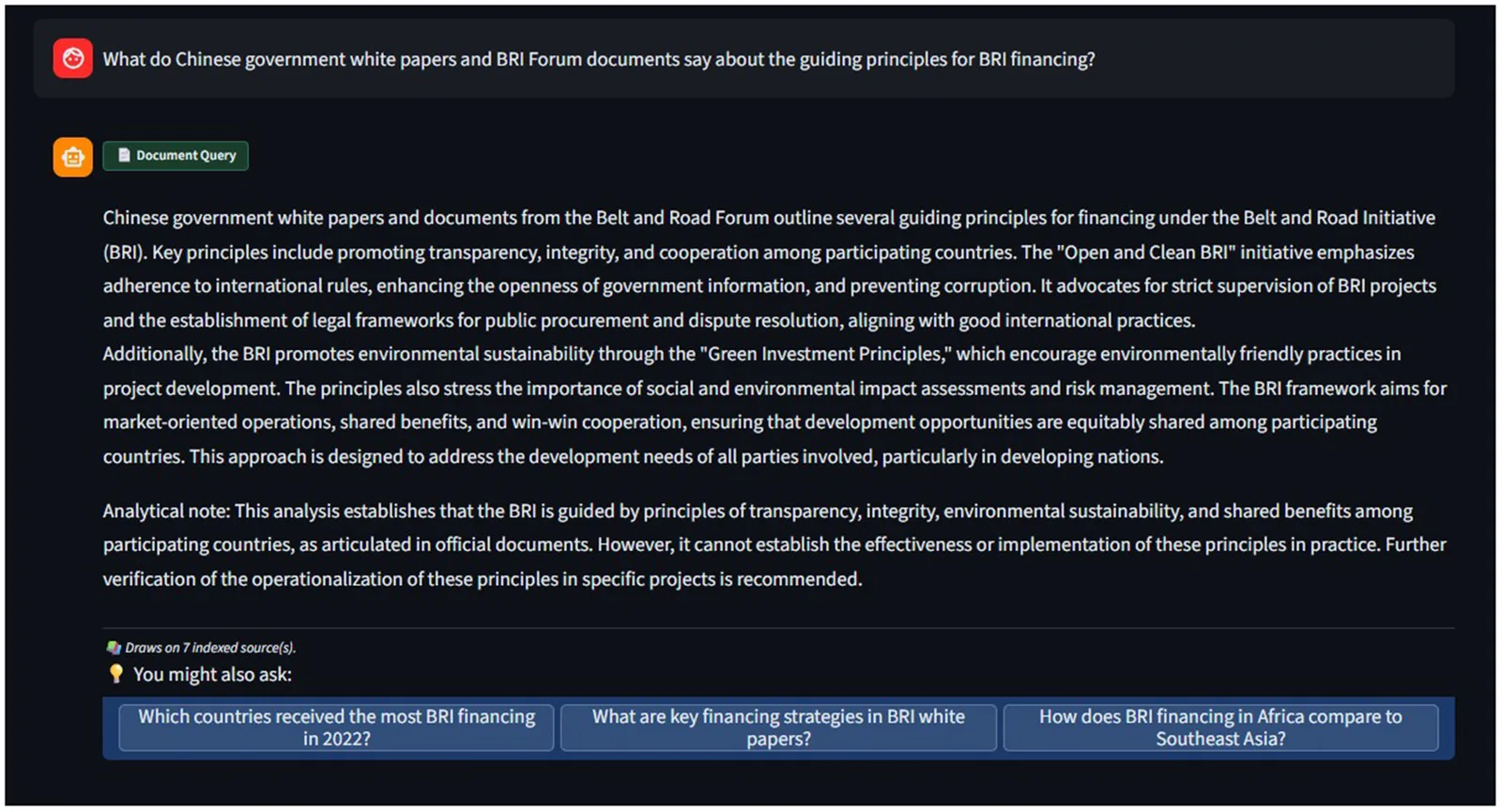
Use Case 2: query + response.

The response demonstrates that document-only retrieval successfully synthesizes official Chinese policy documents while maintaining explicit source attribution (Figure 4). Rather than treating official statements as empirical evidence, the system distinguishes policy commitments from their implementation and recommends further verification where appropriate. The automatically generated follow-up questions also remain within the indexed corpus, illustrating that subsequent interactions are guided by the platform's documented knowledge boundaries rather than unrestricted language-model generation.

### 4.3 Cross-regional comparison (Use Case 3)

*User query:* “Compare Chinese infrastructure financing in Africa vs. South Asia—total commitments, key sectors, and what institutional reports say about debt sustainability risks in each region.”

Multiple hybrid keywords triggered routing to both data and document layers. The AidData results show that Transport and Storage sector dominates in both Africa and South Asia, followed by Energy and Industry, Mining, and Construction, with overall commitment levels higher in South Asia. The debt sustainability synthesis draws on World Bank and IMF documents, attributing concerns that Chinese financing “may lead to increased debt burdens” to those institutional sources rather than presenting them as settled fact. The response notes that institutional reports characterize debt risks as more pronounced in Africa given pre-existing fiscal vulnerabilities, while acknowledging substantial variation within each region.

**Figure 5:**
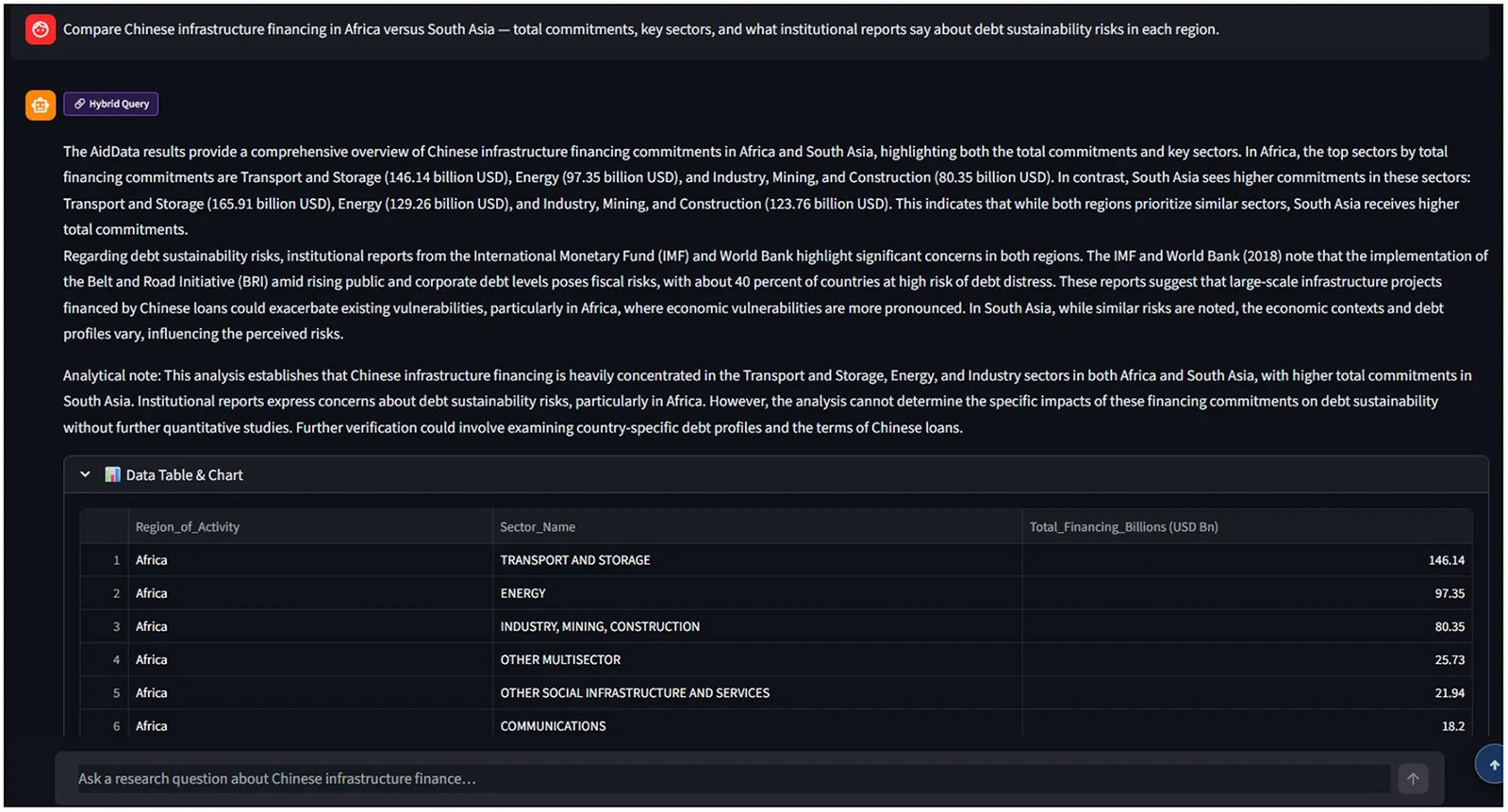
Use Case 3: query + response.

This use case demonstrates the platform's ability to integrate structured financing data with documentary evidence in response to a comparative research question (Figure 5). The quantitative outputs identify regional differences in sectoral financing patterns, while the accompanying synthesis contextualizes these findings using institutional assessments of debt sustainability. Importantly, the analytical note distinguishes descriptive comparisons from causal inference, making clear that regional financing patterns alone cannot establish the causes or consequences of debt outcomes. This illustrates how the platform supports comparative evidence mapping while maintaining explicit methodological boundaries.

Across the three demonstration queries, the routing decisions, retrieved evidence, and synthesized outputs were consistent with the routing architecture described in Section 3.2. The force-hybrid override correctly activated for structured comparative and sector-level questions, while the force-documents override successfully retrieved and synthesized policy documents without unnecessary structured data retrieval. In every case, numerical outputs matched the underlying Data Explorer, retrieved documentary evidence was explicitly attributed, and the analytical notes clearly separated supported findings from questions requiring additional investigation. Together, these demonstrations validate the operational behavior of the routing architecture under representative research scenarios. Systematic benchmarking of this kind is addressed in Section 5.5.

## 5 Discussion

### 5.1 Scalability and extensibility

BRI DataLab currently runs as a single-instance Streamlit application on Streamlit Community Cloud, which suits individual research workflows but is not designed for high-concurrency deployment. Scaling to institutional use would require a dedicated FastAPI backend with connection pooling for DuckDB and rate limiting on LLM calls. The RAG architecture is modular: expanding the corpus requires only re-indexing new documents into ChromaDB with no changes to the routing or synthesis pipeline. Incorporating future AidData releases requires only re-running the harmonization scripts, which are fully documented in the repository.

Future development will also explore replacing proprietary language models with open-weight alternatives as these continue to mature. Recent open-weight models have demonstrated increasingly competitive performance for structured reasoning and retrieval-assisted tasks, making a modular architecture such as BRI DataLab well suited for future migration. While proprietary models currently provide more consistent performance for complex synthesis across structured and unstructured evidence, reducing external API dependence remains an important objective for improving transparency, reproducibility, and long-term sustainability.

### 5.2 What the system enables

BRI DataLab provides reliable analytical leverage on three research tasks. Structured quantitative queries about Chinese infrastructure financing patterns, by country, sector, region, year, or flow type, return accurate results from a harmonized and consistently specified dataset. The self-revision quality check provides an additional automated consistency check to verify that numerical claims in hybrid responses remain aligned with the retrieved structured data. While this does not replace human validation, it reduces the likelihood of inconsistencies between retrieved evidence and synthesized outputs. Documentary retrieval from the curated corpus is grounded in specific source passages with named attributions, so researchers can trace any claim back to its original document. And the hybrid synthesis, when working well, surfaces discrepancies between data patterns and documentary claims rather than flattening them, as Use Case 3 demonstrates. These are meaningful capabilities for a researcher doing initial orientation on a BRI-related question.

### 5.3 Data limitations

The 19% undisclosed amounts problem is a material limitation. For some recipient countries the share of projects with undisclosed financing runs substantially higher, making country-level financial totals systematically incomplete. Figures from BRI DataLab should be read as lower bounds on Chinese infrastructure commitment volumes. The commitment vs. disbursement gap is a separate and equally important constraint and the platform informs researchers what China agreed to finance, not what was built or how much money moved. Disbursement-level data for Chinese overseas finance remains very limited in the public domain, and BRI DataLab does not address this. The dataset's coverage of official state-backed finance also excludes private Chinese investment, informal flows, and sub-sovereign financing, which can be substantial in major BRI economies.

### 5.4 Corpus limitations

The English-language constraint is a structural limitation. Chinese-language primary documentation, including project contracts, inter-ministerial directives, provincial investment plans, and party communications, contains information on BRI implementation that does not appear in the English-language materials. Researchers working on negotiation processes, implementation mechanisms, or domestic Chinese politics will find the document layer limited for these questions. There is also a limitation inherent to semantic retrieval. RAG retrieves passages based on semantic proximity in embedding space, which is not equivalent to analytical relevance. Retrieved passages should therefore not be treated as exhaustive.

A second limitation concerns disciplinary coverage. Although the corpus includes key works on AidData's methodology, debt-trap diplomacy, and the political economy of the Belt and Road Initiative, it does not yet comprehensively represent all major strands of scholarship on Chinese overseas development finance. Future corpus expansion will prioritize additional work on sovereign lending and hidden debt (Horn et al., 2021), alternative development finance datasets and policy-bank lending (Gallagher and Ray, 2020; Moses et al., 2023), political economy perspectives on the Belt and Road Initiative (Jones and Zeng, 2019), and legal and governance dimensions of BRI implementation (Wang, 2019; Chaisse, 2018). Expanding the corpus in this way will strengthen the platform's ability to support interdisciplinary research while maintaining transparent corpus boundaries and explicit source attribution.

### 5.5 AI and methodological limitations

Language models produce fluent, syntactically confident text regardless of whether the underlying information is reliable. The system prompt's epistemic constraints reduce but do not eliminate the risk of plausible-sounding but incorrect outputs. The self-revision step adds an additional automated consistency check but is itself an LLM, not a human expert. Although the three validation cases presented in Section 4 demonstrate that the routing architecture functions as intended across structured, document-only, and hybrid query pathways, they do not constitute a systematic quantitative evaluation of routing performance. Future work will include a dedicated benchmarking study using a representative evaluation dataset to assess routing accuracy, retrieval quality, and synthesis reliability across different query categories. Such an evaluation would provide quantitative evidence for the platform's performance while complementing the qualitative validation presented in this paper. Numerical accuracy is the highest-risk category: financial figures quoted in hybrid responses should be verified against the Data Explorer before being cited in research outputs. BRI DataLab is an exploratory research tool. Its outputs are starting points for research, not findings.

Response latency varies according to query complexity rather than query length alone. Queries that trigger multiple routing layers together with extensive document retrieval and synthesis require additional processing time compared with straightforward structured or document-only requests. The latency values reported in Table 3 therefore represent typical operating conditions rather than fixed execution times. Future optimization will focus on caching frequently executed queries, improving retrieval efficiency, and reducing synthesis overhead for complex analytical requests.

### 5.6 A generalizable design principle

The constraint architecture described here has application beyond this specific tool. AI-assisted research interfaces in IR and political science should be constrained at the system level to prohibit what they cannot reliably establish. The specific constraints documented here, source-only claims, attribution of contested arguments, mandatory commitment vs. disbursement distinction, causal language prohibition, and analytical notes on every response, are transferable design decisions. The primary risk in AI-assisted research is not that scholars will mistake AI outputs for primary sources. It is that AI outputs will flatten the evidential texture of contested empirical debates into the confident register of settled knowledge. Designing against this requires explicit constraints built into the system, not trust in the model's general reliability (Grimmer and Stewart, 2013). This design logic is consistent with broader AI-governance scholarship that emphasizes embedding transparency, accountability, and oversight into the design and deployment of AI systems so that they can better safeguard user wellbeing and remain open to scrutiny (Cheong, 2024).

## 6 Conclusion

BRI DataLab's contribution is integration. AidData datasets are publicly available. Chinese government policy documents are on government portals. The peer-reviewed BRI literature is accessible through standard academic channels. What BRI DataLab does is bring these three layers into a single research interface, governed by methodological decisions that are documented here and open to scrutiny. By harmonizing tranche-level records into a clean project-level dataset, constructing a document corpus with explicit and defensible inclusion criteria, and designing an AI system that attributes contested claims and excludes causal inference without quantitative support, the platform gives researchers something no individual source layer provides, the ability to move between financing data, official discourse, and scholarly synthesis within a single query, while keeping a clear record of where each element comes from and what it cannot establish.

For researchers working on Chinese development finance, this reduces the setup cost of preliminary analysis considerably. For instance, a researcher examining Pakistan's energy infrastructure commitments can retrieve the AidData figures, the Chinese government's official framing of energy cooperation, and the GFDC reports' assessment of green finance trends in a single hybrid query, and receive a response that distinguishes these three source types and states clearly what the synthesis cannot claim. That is more useful than any individual source alone, and more honest about its limitations than an unstructured search would be.

Future development priorities extend beyond expanding the platform's technical capabilities. Although the validation presented in this paper demonstrates that the routing architecture, retrieval mechanisms, and synthesis pipeline function as intended, it does not evaluate how researchers interact with the platform in practice. A formal user evaluation involving researchers and policy analysts will therefore form an important next stage of development, examining whether the platform improves research efficiency, transparency, and evidence discovery during real analytical workflows. Additional priorities include expanding the corpus to include relevant Chinese-language primary documentation and incorporating disbursement-level information as it becomes publicly available.

The broader argument is direct, transparent AI-assisted research infrastructure is a legitimate and, in data-intensive subfields, a necessary contribution to computational social science. What makes it legitimate is not the complexity of the technology. It is the explicitness of the methodological decisions, what data was included and why, what documents were selected and by what criteria, and what the AI was designed to prevent as much as what it was designed to enable, and this paper documents all three.

## Data Availability

The study draws on publicly available data sources, including AidData's China's Global Loans and Grants Dataset v1.0 and publicly available policy documents and institutional reports. No original dataset was generated for public deposit. Processed data used in the analysis are available from the author upon reasonable request. The source code for BRI DataLab is publicly available at: https://github.com/sanoop-ssk/bri-datalab
